# Microbial Communities in and Around the Siboglinid Tubeworms from the South Yungan East Ridge Cold Seep Offshore Southwestern Taiwan at the Northern South China Sea

**DOI:** 10.3390/microorganisms12122452

**Published:** 2024-11-28

**Authors:** Yin Li, Zhiwei Ye, Mei-Chin Lai, Char-Shine Liu, Charles K. Paull, Saulwood Lin, Shu-Jung Lai, Yi-Ting You, Sue-Yao Wu, Chuan-Chuan Hung, Jiun-Yan Ding, Chao-Jen Shih, Yen-Chi Wu, Jingjing Zhao, Wangchuan Xiao, Chih-Hung Wu, Guowen Dong, Hangying Zhang, Wanling Qiu, Song Wang, Sheng-Chung Chen

**Affiliations:** 1 School of Resources and Chemical Engineering, Sanming University, Sanming 365004, China; lijiang413508@126.com (Y.L.); 15294566095@163.com (Z.Y.); zhaojj191scus@163.com (J.Z.); xwc@fjsmu.edu.cn (W.X.); chihhung@yeah.net (C.-H.W.); gwdong2008@163.com (G.D.); 18005985018@163.com (H.Z.); wanning084020@163.com (W.Q.); 19154084327@163.com (S.W.); 2 Fujian Provincial Key Laboratory of Resources and Environmental Monitoring and Sustainable Management and Utilization, Sanming University, Sanming 365004, China; 3 College of Environment and Safety Engineering, Fuzhou University, Fuzhou 350108, China; 4 Department of Life Sciences, National Chung Hsing University, Taichung 402202, Taiwan; sjlai01@gmail.com (S.-J.L.); snoopy7117@gmail.com (Y.-T.Y.); sywu.kiki@gmail.com (S.-Y.W.); chuanchuanhung@gmail.com (C.-C.H.); jiunyanding@gmail.com (J.-Y.D.); 5 Institute of Oceanography, National Taiwan University, Taipei 106319, Taiwan; csliu@ntu.edu.tw (C.-S.L.); swlin@ntu.edu.tw (S.L.); 6 Monterey Bay Aquarium Research Institute, Moss Landing, CA 95039-9644, USA; paull@mbari.org; 7 Graduate Institute of Biomedical Sciences, China Medical University, Taichung 406040, Taiwan; 8 Research Center for Cancer Biology, China Medical University, Taichung 406040, Taiwan; 9 Bioresource Collection and Research Center, Food Industry Research and Development Institute, Hsinchu 300193, Taiwan; cjs23@firdi.org.tw (C.-J.S.); ycw@firdi.org.tw (Y.-C.W.); 10 College of Chemistry and Materials Science, Fujian Normal University, Fuzhou 350117, China; 11 Medical Plant Exploitation and Utilization Engineering Research Center, Sanming University, Sanming 365004, China

**Keywords:** South Yungan East Ridge, *Paraescarpia*, anerobic methane-oxidizing archaea, microbial mat, cold seep

## Abstract

To date, only a few microbial community studies of cold seeps at the South China Sea (SCS) have been reported. The cold seep dominated by tubeworms was discovered at South Yungan East Ridge (SYER) offshore southwestern Taiwan by miniROV. The tubeworms were identified and proposed as *Paraescarpia formosa* sp. nov. through morphological and phylogenetic analyses. The endosymbionts in the trunk of *P. formosa* analyzed by a 16S rRNA gene clone library represented only one phylotype, which belonged to the family Sedimenticolaceae in Gammaproteobacteria. In addition, the archaeal and bacterial communities in the habitat of tubeworm *P. formosa* were investigated by using high-phylogenetic-resolution full-length 16S rRNA gene amplicon sequencing. The results showed that anerobic methane-oxidizing archaea (ANME)-1b was most abundant and ANME-2ab was minor in a consortia of the anerobic oxidation of methane (AOM). The known sulfate-reducing bacteria (SRB) partners in AOM consortia, such as SEEP-SRB1, -SRB2, and -SRB4, *Desulfococcus* and *Desulfobulbus*, occurred in a small population (0–5.7%) at the SYER cold seep, and it was suggested that ANME-1b and ANME-2ab might be coupled with multiple SRB in AOM consortia. Besides AOM consortia, various methanogenic archaea, including Bathyarchaeota (Subgroup-8), Methanocellales, Methanomicrobiales, Methanosarcinales, Methanofastidiosales and Methanomassiliicoccales, were identified, and sulfur-oxidizing bacteria *Sulfurovum* and *Sulfurimonas* in phylum Epsilonbacteraeota were dominant. This study revealed the first investigation of microbiota in and around tubeworm *P. formosa* discovered at the SYER cold seep offshore southwestern Taiwan. We could gain insights into the chemosynthetic communities in the deep sea, especially regarding the cold seep ecosystems at the SCS.

## 1. Introduction

Cold seeps are the regions of the ocean floor where hydrogen sulfide, methane and other hydrocarbon-rich fluid seepage occurs. At cold seeps, microbial chemosynthetic carbon fixation is the basis for the food web, and those metabolic processes driven by microorganisms, such as methanogenesis, the anerobic oxidation of methane (AOM), sulfate reduction, the aerobic oxidation of methane, and sulfide oxidation, have been identified [1,2,3,4]. Like hydrothermal vents, cold seeps hosting tubeworms, extensive mussel and clam beds, and dense shrimp and crab aggregations are also reliant on symbionts that use chemical energy to fix organic carbon [5].

Microbially mediated AOM is a key process in the regulation of methane emissions to the atmosphere [6]. The consortia of anerobic methane-oxidizing archaea (ANME) and sulfate-reducing bacteria (SRB) couples the anerobic oxidation of methane to sulfate reduction [6]. Three different groups of ANME have been identified. ANME-1, including ANME-1a, -b and ANME-1 Guaymas, forms a discrete phylogenetic group, which may represent a new order within the class Methanomicrobia. ANME-2a, -2b, -2c, and -2d and ANME-3 fall into the methanogenic order Methanosarcinales in the class Methanomicrobia [4,6,7,8]. In addition, several SRB members affiliated with Deltaproteobacteria, such as *Desulfosarcina*/*Desulfococcus* (DSS) subclade SEEP-SRB1, SEEP-SRB2, *Desulfofervidus* lineages and some members of the genus *Desulfobulbus,* have been identified in syntrophic methane-oxidizing consortia in these environments [9,10,11,12,13]. AOM is widely considered to be the main process occurring in cold seep sediments due to the ubiquitous ANME sequences in 16S rRNA gene surveys and the microscopic detection of the striking aggregates of ANME and SRB [14].

Siboglinids are tube-dwelling annelids that are important members of deep-sea chemosynthetic communities, including cold seeps, hydrothermal vents, whale falls, wood falls, and reduced sediments [15,16,17]. Due to being mouthless and gutless in the adult period, tubeworms within the annelid family Siboglinidae rely on chemosynthetic bacterial symbionts to provide them with organic compounds and nutrition. The worm acquires oxygen, sulfide, and carbon dioxide from seeps or vent fluids mixed with seawater and delivers them to the symbiotic bacteria for sulfide oxidation and autotrophy. The endosymbiotic bacteria are known to localize in the trunk region of tubeworms, within the specialized organ called the trophosome [16,18]. The energy produced by symbiotic bacteria provides carbon for growth and metabolism to the worms [19]. The endosymbionts of vestimentiferans inhabiting sulfide-rich hydrothermal vents are monospecific for their host [20,21]. However, previous studies suggest that vestimentiferans of methane-rich seeps may harbor multispecific symbionts [22,23,24]. Recently, dual symbiosis with co-occurring sulfur-oxidizing symbionts in tubeworm *Lamellibrachia anaximandri* from a Mediterranean hydrothermal vent has been verified [25].

The South China Sea (SCS) is a marginal sea in the convergence zone between the Pacific plate, the Eurasian plate and the Australian plate [26]. More than 40 cold seeps have been found and recorded on both the northern and southern continental margins of the SCS [27]. However, only a few studies of microbial communities of SCS cold seeps have been reported, which include Haiyang cold seep [28], Haima cold seep [29], Jiaolong cold seep [30] and two cold seeps at gas hydrate drilling Site GMGS2-08 [31]. The aim of our project is to investigate and compare the archaeal and bacterial communities in cold seep sediments or gas hydrate-bearing areas offshore southwestern Taiwan at the northern SCS. In the present study, we discovered a cold seep dominated by tubeworms at the South Yungan East Ridge (SYER) area offshore southwestern Taiwan. The method of Pacific Biosciences (PacBio) near full-length 16S rRNA gene amplicon sequencing, which provides higher taxonomic resolution at the species level [32,33], was applied to study the archaeal and bacterial communities in the SYER cold seep. In addition, the compositions of ecto- and endo-symbionts of the cold-seep vestimentiferan tubeworm *Paraescarpia* sp. isolated from SYER were also analyzed by using 16S rRNA gene clone-based Sanger sequencing.

## 2. Materials and Methods

### 2.1. Study Area, Sample Collection and Geochemical Analysis

**Figure 1:**
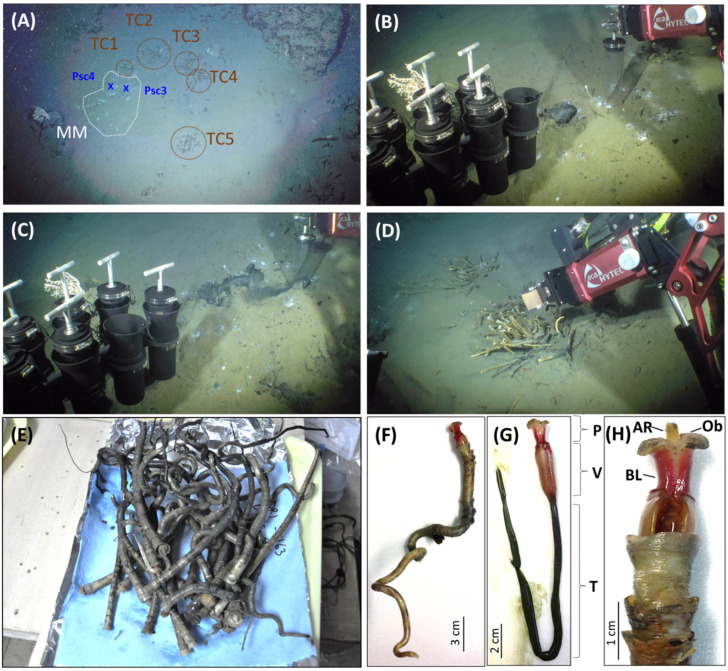
The photographs show the sampling sites, sampling process of the microbial mat and tubeworms, and views of tubeworms. (**A**) Overview of the sampling site. TC1~5, tubeworm clusters #1~5; MM, microbial mat; x, sampling sites for Psc3 and Psc4. The sampling photos of the push core Psc4 (**B**), Psc3 (**C**) and tubeworms (**D**). Photo of tubeworms right after collection (**E**). Overview of the tubeworm ORI-1163B-Tw1 with (**F**) or without (**G**) the external tube. Close view of the anterior part tubeworm (**H**). P, plume; V, vestimentum; T, trunk; AR, axial rod; Ob, obturaculum; BL, branchial lamellae.

Widely and intensively distributed bottom simulating reflectors (BSRs) imply the high potential occurrence of gas hydrate offshore southwestern Taiwan [34]. In order to investigate the potential drilling sites of gas hydrates, the joint cruises (ORI-1163B&C cruises) of the R/V Ocean Researcher I and Monterey Bay Aquarium Research Institute (MBARI) AUV-miniROV were implemented on 7–20 May 2017. During the survey at the SYER region (Figure S1, water depth, 1237 m; location at 119.8694 E, 22.1532 N) by miniROV, five clusters of tubeworms were found nearby the white spots of a microbial mat (Figure 1A). Two push core sediments (push cores Psc4, Figure 1B; Psc3, Figure 1C) and dozens of tubeworms (Figure 1D,E) were collected at this site by miniROV on 8 May 2017. The distance between Psc3 and Psc4 was around 50 cm. The Psc3 (core length, 12 cm) was used to analyze pore water-dissolved sulfide and sulfate concentrations. Sediment samples from push cores were immediately sliced every 2 cm into polyethylene (PE) centrifuge tubes and the pore water from sliced sediments was obtained from centrifugation and filtration. Dissolved sulfide concentrations were measured by the absorbance of methylene blue complex at 670 nm on board [35]. The remaining pore water was stored in PE vials and sulfate concentrations were determined in a shore-based laboratory by ion chromatography with a Dionex 4500i ion chromatograph (Thermo Fisher Scientific, Sunnyvale, CA, USA) equipped with a conductivity detector and an IonPacAS4A anion exchange column (Thermo Fisher Scientific, Sunnyvale, CA, USA) [36]. The Psc4 (core length, 14 cm) was selected to extract DNA for archaeal and bacterial community analyses.

### 2.2. Cloning, Sequencing and Phylogenetic Analysis

The DNA samples from different organs of tubeworm #1 (named as ORI-1163B-Tw1), including the axial rod, obturaculum, and trunk, were extracted according to the instruction of the Genomic DNA Mini Kit (Tissue, Geneaid Biotech Ltd., New Taipei City, Taiwan) (Figure 1F–H). The primer sets for polymerase chain reaction (PCR) amplifications of the cytochrome C oxidase subunit I (COI) gene, 18S rRNA gene, and bacterial 16S rRNA gene are listed in Table S1. The conditions for PCR amplification were according to the manual of Blend Taq^®^ Plus (TOYOBO Co., Ltd., Osaka, Japan). The cloning was constructed according to the technical manual of pGEM^®^-T vector (Promega, Madison, WI, USA). Sequencing was performed by Mission Biotech Co., Ltd. (Taipei, Taiwan) using the ABI 3730XL DNA Analyzer (Applied Biosystems^TM^, Thermo Fisher Scientific, Waltham, MA, USA). The related COI genes, 18S rRNA genes and 16S rRNA genes used in this study were obtained from the GenBank database, NCBI Reference Sequence Database or SILVA database. Phylogenetic trees were reconstructed by using the MEGA7 program [37] using Maximum Likelihood or Neighbor-Joining methods with 1000 bootstrap replicates. The DDBJ/EMBL/GenBank accession numbers for the sequences obtained from clone libraries in this study are the tubeworm ORI-1163B-Tw1 COI gene (MH459063), 18S rRNA gene (MH464138), and 16S rRNA gene sequences of bacterial symbionts found in the axial rod (MH455345-MH455360), obturaculum (MH454643-MH454659) and trunk (MH454580-MH454596).

### 2.3. PacBio 16S rRNA Gene Amplicon Sequencing and Analysis of Microbial Communities

The environmental DNA samples from three selected depths (2–4, 6–8 and 10–12 cm below the seafloor, cmbsf) of sediment layers of the push core Psc4 were extracted according to the handbook of the DNeasy^®^ PowerMax^®^ Soil Kit (Qiagen, Hilden, Germany). The primer pairs for the archaeal and bacterial V1-V9 16S rRNA gene are listed in Table S1. The amplified DNA was sequenced in Genomics Biotech Co., Ltd. (Taipei, Taiwan) by using the single-molecule real-time (SMRT) PacBio sequencing technology with the PacBio Sequel^TM^ system (Pacific Biosciences, Menlo Park, CA, USA) according to the standard manufacturer’s condition. SMRT bell library prep and sequencing used the currently available reagent kits Template Preparation 3.0, Polymerase Binding P6 and Sequencing Chemistry C4.

Raw reads were processed by SMRTLink software v5.1.0 to obtain demultiplexed consensus sequences. Sequence data were processed using the software package QIIME version 1.80 [38]. Sequences shorter than 1000 nt and longer than 2000 nt were removed prior to downstream analyses. Chimeric reads were filtered out by using Mothur software v.1.33.3 [39]. The final effective reads were used for taxonomic assignment against the SILVA database (SILVA release 132) using the SILVAngs analysis platform [40,41,42]. After SILVAngs classification, the non-target classified sequences, e.g., bacteria and eukarya sequences in archaea datasets, were removed for further community analysis (Table S2). The effective reads were also processed by ChunLab’s 16S Microbiome Profiling Service with the EzBioCloud database to have more comprehensive species-level profiling (Table S3) [43]. The clean reads of 16S rRNA gene amplicon sequencing were submitted to NCBI under BioProject PRJNA490800.

## 3. Results and Discussion

### 3.1. The Tubeworm ORI-1163B-Tw1 Was Proposed as Paraescarpia Formosa

**Figure 2:**
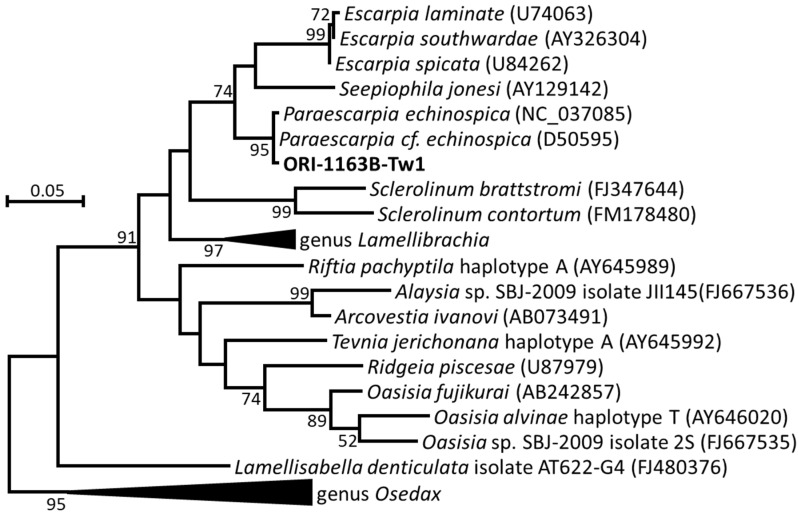
Molecular phylogenetic analysis of the tubeworm ORI-1163B-Tw1 and related species based on COI gene sequences. The evolutionary history was inferred by using the Maximum Likelihood method based on the Kimura 2-parameter model [47] and evolutionary analyses were conducted in MEGA7 [37]. Numbers at the nodes indicate the proportion of occurrences in 1000 bootstrap replicates. The scale represents 0.05 substitutions per nucleotide site.

The morphology and structure of the tubeworm ORI-1163B-Tw1 (Figure 1D–H) is similar to deep-sea tubeworms *Paraescarpia echinospica* [44], *Seepiophila jonesi* [45], and *Escarpia southwardae* [46] but shows the significant differences among the structures of the plume, vestimentum and external tube to these species. The axial rod of the plume and vestimentum of the tubeworm ORI-1163B-Tw1 (like a cylinder, Figure 1H) are different to those of *E. southwardae* (like an acicula) [46]. And the external tube structure of the tubeworm ORI-1163B-Tw1 has two contiguous three collars (Figure 1F), which is different to *P. echinospica* (three separate collars) [44] and *S. jonesi* (one contiguous two–three collars) [45]. A phylogenetic analysis of COI gene sequences revealed that the tubeworm ORI-1163B-Tw1 is more closely related to the deep-sea tubeworm *P. echinospica* (99.7–99.8% sequence similarity) and shares 90.4% and 88.5–88.9% similarities with *S. jonesi* and species in the genus *Escarpia*, respectively (Figure 2). Based on its distinct morphological differences, the tubeworm ORI-1163B-Tw1 may represent a new species under the genus *Paraescarpia*, and the name “*Paraescarpia formosa* sp. nov.” is proposed.

The phylogenetic analyses of the concatenated mitochondrial genes strongly support a sister relationship between genera *Paraescarpia*, *Seepiophila* and *Escarpia* [48], but the 18S rRNA gene sequences among these tubeworms do not show a divergence for species and genus delineation (Figure S2). The first and only one valid species in the genus *Paraescarpia* was *Paraescarpia echinospica*, which was found at sediments near mussel clump, Edison Seamount, Lihir Island [44]. *Paraescarpia* specimens also have been found in the cold seeps of Papua New Guinea, Nankai Trough and Haima, as well as the hydrothermal vents of the Okinawa Trough [49,50]. And these tubeworms observed and collected in the ORI-1163B cruise at the SYER cold seep area offshore southwestern Taiwan are the first record of the genus *Paraescarpia* in Taiwan.

### 3.2. The Sedimenticolaceae Phylotype Was Identified as a Sulfur-Oxidizing Endosymbiont in the Trunk

**Figure 3:**
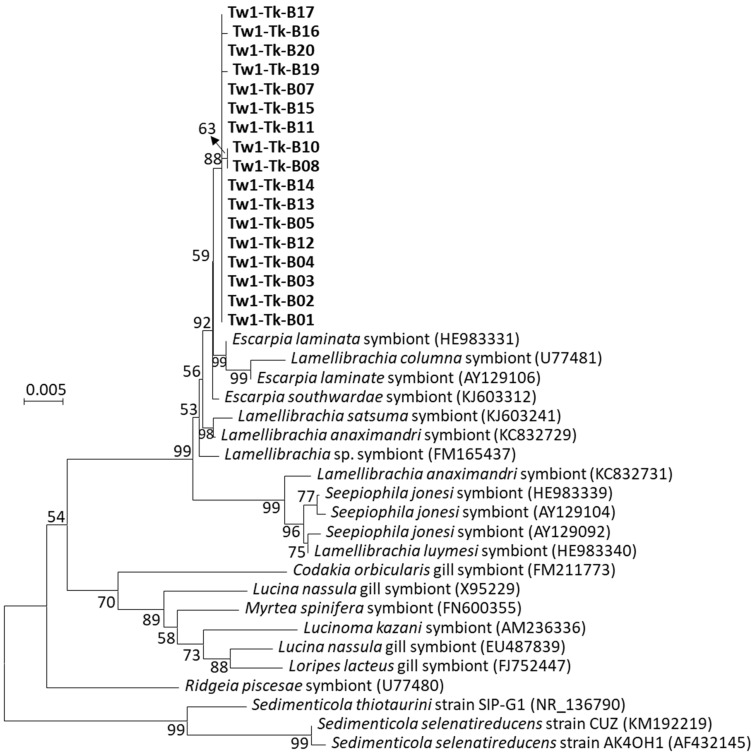
Phylogenetic analysis of SSU rRNA genes of bacterial symbionts from the trunk of tubeworm *P. formosa* ORI-1163B-Tw1 and other related sequences. The evolutionary history was inferred by using the Neighbor-Joining method, and evolutionary analyses were conducted in MEGA7 [37]. Numbers at the nodes indicate the proportion of occurrences in 1000 bootstrap replicates. Symbiont clones from the trunk of the tubeworm ORI-1163B-Tw1 are represented in boldface. The scale represents 0.005 substitutions per nucleotide site.

In order to investigate the endosymbiotic microbiota in the trunk of tubeworm *P. formosa* ORI-1163B-Tw1, the total DNA of the tissue fluid inside the trunk was extracted as a template for archaeal and bacterial 16S rRNA gene amplification. No PCR product of the archaeal 16S rRNA gene was obtained. Seventeen bacterial 16S rRNA gene clones from the trunk of *P. formosa* ORI-1163B-Tw1 shared 99.83–100% similarity with each other, which indicated that these clones represented one phylotype that belonged to the family Sedimenticolaceae in Gammaproteobacteria by SILVA database classification. These symbiont clones were phylogenetically analyzed with the symbionts of vestimentiferan tubeworms, bivalves and cultured *Sedimenticola* strains (Figure 3). Phylogenetic analysis revealed that symbionts of *P. formosa* ORI-1163B-Tw1 more closely clustered with other symbionts found in cold seep or vent tubeworms *Escarpia* (99.73–99.86%), *Lamellibrachia* (98.04–99.59%) and *Seepiophila* (97.78–97.96%) species. The symbionts of *P. formosa* ORI-1163B-Tw1 were ~95% similar with bivalves’ symbionts and shared 93–94% similarity with cultivated representative sulfur-oxidizing autotrophs *Sedimenticola thiotaurini* [51] and *S. selenatireducens* [52]. The results suggested that *P. formosa* ORI-1163B-Tw1 harbored one phylotype and monospecific sulfur-oxidizing endosymbionts that occupied the trophosome of the trunk and obtained oxygen, sulfide, and carbon dioxide from their host to convert into organic compounds and nutrition back to their host. Furthermore, high similarities between the symbiotic bacteria of *P. formosa* ORI-1163B-Tw1, *Escarpia* and *Lamellibrachia* tubeworms supported that vestimentiferan tubeworms acquire their symbionts through horizontal transmission from the surrounding environment [53]. Indeed, the PacBio near full-length 16S rRNA gene sequencing reads that belong to the family Sedimenticolaceae were found throughout all three sampling depths in push core Psc4, and more were found in shallower (2–4 cmbsf) sections (Figure S3).

### 3.3. Potential Sulfur-/Methane-Oxidizing Symbionts Were Found in the Axial Rod and Obturaculum

**Figure 4:**
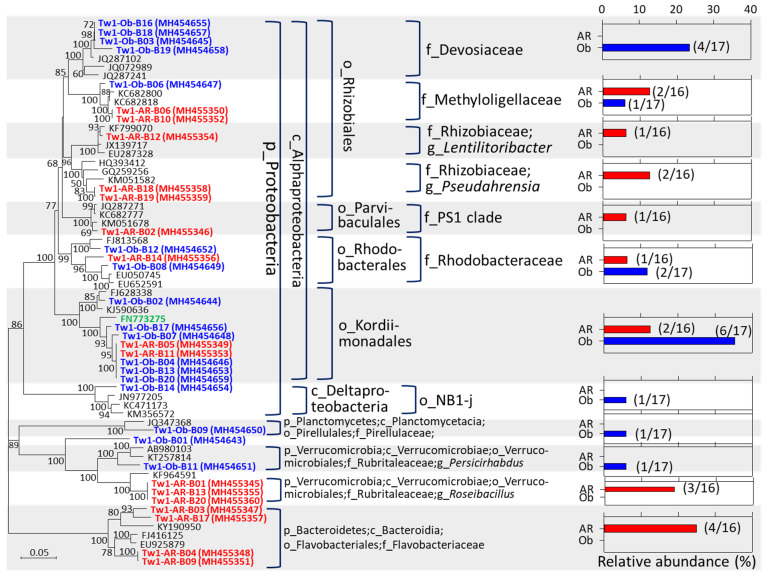
A phylogenetic analysis of bacterial symbiont 16S rRNA gene sequences from the axial rod and obturaculum of *P*. *formosa* ORI-1163B-Tw1. The SILVA classification results and relative abundance of each cloned phylotype are shown. Ratios of the number of each cloned phylotype to total clone number are shown in parentheses. Symbiont clones from the axial rod (AR) and obturaculum (Ob) of the tubeworm ORI-1163B-Tw1 are represented in red and blue colors, respectively. Green color-labeled sequence: FN773275, the bacterium endosymbiont of the *Osedax mucofloris* partial 16S rRNA gene, clone Omu 3 c112.

The bacterial symbiont clones in the axial rod were classified into Alphaproteobacteria (9/16 clones = 56.25%), Bacteroidetes (4/16 clones = 25%), and Verrucomicrobia (3/16 clones = 18.75%) (red color in Figure 4). In the obturaculum, symbiont clones belonged to Alphaproteobacteria (14/17 clones = 82.35%), Planctomycetes (1/17 clones = 5.88%), and Verrucomicrobia (1/17 clones = 5.88%), and 1 was unclassified (blue color in Figure 4). The symbiont phylotypes that co-occurred in both the axial rod and obturaculum belonged to the family Rhodobacteraceae, the order Kordiimonadales, the family Rubritaleaceae, and the family Methyloligellaceae. The Rhodobacteraceae sequences have been identified as the dominant symbiont in the brittle star *Amphipholis squamata* [54] and the coral *Astrangia poculata* [55]. In addition, the members of Rhodobacteraceae are the key players of the microbial community of the initial biofilm formed in Eastern Mediterranean coastal seawater [56].

The order Kordiimonadales was proposed by a marine bacterium, *Kordiimonas gwangyangensis*, isolated from marine sediments of Gwangyang Bay, the Republic of Korea, which was capable of degrading high-molecular-mass polycyclic aromatic hydrocarbons [57]. The symbiont clones of the order Kordiimonadales were situated at the same clade with the sequence (FN773275) from the bacterium endosymbiont of *Osedax mucofloris* [58]. And the free-living species *Rubritalea spongiae* within the family Rubritaleaceae was isolated from a marine sponge [59]. Furthermore, the symbiont clones belonging to the family Devosiaceae were more closely related with uncultured sequences (JQ287102 and JQ287241) obtained from the inactive hydrothermal sulfides [60], which suggested that these clones represented sulfide-oxidizing bacteria. Based on the SILVA database, the family Methyloligellaceae contains three genera, including *Methyloceanibacter*, *Methyloligella*, and *Rhodobium*. Cultured representatives in the genera *Methyloceanibacter* and *Methyloligella* are methylotrophs, and notably, the *Methyloceanibacter methanicus* strain R-67174^T^ was capable of oxidizing methane as sole source of carbon and energy [61,62,63]. Taken together, the symbionts detected in both the axial rod and obturaculum of the tubeworm *P. formosa* ORI-1163B-Tw1 may play roles in sulfur, methanol, methylamine or methane utilizations.

### 3.4. ANME-1 Was Dominant in AOM Consortia at the SYER Cold Seep

**Figure 5:**
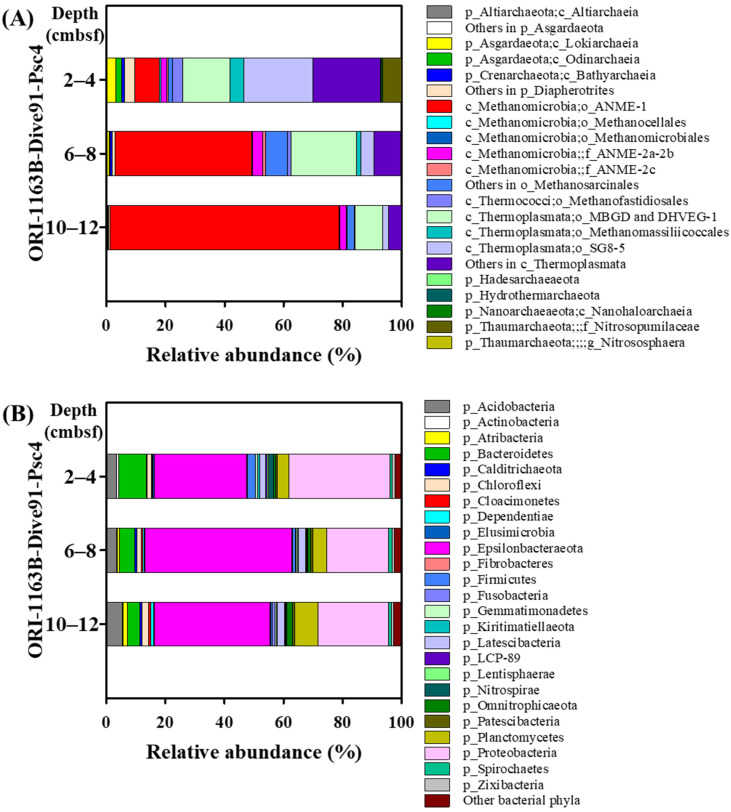
The taxonomic distribution of the prokaryotic communities identified in three sampling depths of 2–4, 6–8, and 10–12 cm below the seafloor (cmbsf) of the push core ORI-1163B-Dive91-Psc4. The 16S rRNA gene amplicons were classified by comparing them with the SILVA 132 database. (**A**) Archaeal community. (**B**) Bacterial community. Abbreviations: p, phylum; c, class; o, order; f, family; g, genus.

The major archaeal phylum in the push core Psc4 is Euryarchaeaota, which presents 83.2–98.7% relative abundance through three sampling depths (Figure 5A). Within the phylum Euryarchaeota, the anerobic methane-oxidizing archaea ANME-1, mainly ANME-1b, significantly increased in abundance (from 8.44 to 77.72%) at depths from 0–2 cmbsf to 10–12 cmbsf, whereas both orders Methanomassiliicoccales and SG8-5 in the class Thermoplasmata decreased from 4.74 to 0.18% and 23.52 to 1.96%, respectively (Figure 5A). Similarly, the relative abundances of both Lokiarchaeia and Odinarchaeia in Asgardaeota and Diapherotrites also gradually decreased with the depth (Figure 5A; Table S4). ANME-2 groups occurred in minor abundance at all sampling depths (2.35–4.33%). The high relative abundance of ANME-1b suggested that the AOM reaction is highly active here. The AOM has been identified as the major pathway of methane consumption in various cold seep ecosystems and in releasing sulfide and bicarbonate into the pore water [64]. The sulfate concentration of pore water in the push core Psc3 was 24 mM at ~1 cm below the seafloor (cmbsf) and decreased sharply with the depth increasing (Figure S4). Then, the sulfate concentration was remained ~12.5 mM below 8 cmbsf. Inversely, the dissolved sulfide appeared at the depth of 1 cm and then increased rapidly to 3.2 mM at 5 cmbsf and leveled off (Figure S4). The elevated fluxes of sulfide, the reaction product of AOM consortia, might support unique chemosynthetic seep communities as the tubeworm communities present at the SYER cold seep.

### 3.5. Various Methanogenic Archaea Were Found at the SYER Cold Seep

Besides ANME-1 and ANME-2, various methanogenic archaea were identified in this sampling site, including Bathyarchaeota (0.21–1.04%), Methanocellales (0.08–0.47%), Methanomicrobiales (0.02–0.09%), Methanosarcinales (1.58–7.27%), Methanofastidiosales (0.38–3.41%), and Methanomassiliicoccales (0.18–4.74%) (Table S4), which may indicate that the methane-producing communities were also highly active here. The members of Bathyarchaeota have wide metabolic capabilities, including acetogenesis, methane metabolism, dissimilatory nitrogen and sulfur reduction and have been clustered into 25 subgroups based on 16S rRNA gene phylogenetic analysis [65,66,67,68,69,70,71,72,73]. In order to investigate the potential metabolic capabilities of bathyarchaeotal reads identified in this study, phylogenetic trees of bathyarchaeotal 16S rRNA genes were constructed, which included the sequences obtained from this study and the bathyarchaeotal sequences from the previous publication [65]. The results indicated that the bathyarchaeotal sequences derived from this study were grouped with Subgroups-5a, -8, -15, -17, and -23 and unclassified (Figure S5). One bathyarchaeotal genome bin BA2, which belongs to Subgroup-8, contains methyl-coenzyme M reductase (MCR)-encoding genes and additional genes of typical methane metabolism, reflecting similar methylotrophic methanogenesis activity [66]. Therefore, it was suggested that the Subgroup-8 bathyarchaeotal sequences from this study might represent the methanogenic archaea.

In addition, through the analysis of 16S Microbiome Pipeline on the EZBioCloud website, some reads were taxonomically classified at the species level as known cultured representatives, such as hydrogenotrophic *Methanoregula boonei* and *Methanoregula formicica*, methylotrophic *Methanococcoides alaskense* and *Methanococcoides vulcani*, and acetotrophic *Methanosarcina vacuola*, *Methanosarcina acetivorans* and *Methanosaeta concilii* (Figure S6). Among them, *Methanococcoides alaskense*, which was firstly isolated from the sulfate-reducing zone of the sediments in Skan Bay, Alaska [74], was relatively abundant and widely distributed at three sampling depth ranges in the push core (Figure S6).

### 3.6. Both Genera Sulfurovum and Sulfurimonas Were Dominant at the SYER Cold Seep

The newly proposed new phylum Epsilonbacteraeota (31.38 to 49.65%) [75] was most abundant in the bacterial community around the habitat of *P. formosa* ORI-1163B-Tw1, followed by phyla Proteobacteria (21.07 to 34.24%), Bacteroidetes (4.27 to 9.24%), Plantomycetes (3.85 to 7.89%), and Acidobacteria (3.34 to 5.46%) through three sampling depths in the push core Psc4 sediments (Figure 5B; Table S5). Within the phylum Epsilonbacteraceota, both genera *Sulfurovum* and *Sulfurimonas* are dominant in this tubeworm’s habitat. The relative abundance of the genus *Sulfurovum* increased with the depth increasing (5.69 to 27.68%); however, the abundance of the genus *Sulfurimonas* presented an inverse correlation (23.19 to 9.36%) (Table S5). Most cultivated representatives of *Sulfurovum* and *Sulfurimonas* were isolated from hydrothermal fields and described as sulfur-, thiosulfate-oxidizing chemolithoautotrophs [76,77,78,79,80,81,82,83]. Sulfide from hydrothermal vents and cold seeps is one of the most abundant substrates to various sulfur-oxidizing microorganisms. Evidently, the profile of the vertical distribution of the genus *Sulfurovum*, but not *Sulfurimonas*, was similar to the distribution profile of sulfide, potentially due to the ANME-1b-dominated AOM reaction (Figure S4).

Besides the gradually downward increasing abundance of the genus *Sulfurovum*, some minor phylogenetic groups also presented similar profiles, including classes Phycisphaerae (2.35 to 6.62%, within the phylum Planctomycetes) and Aminicenantia (0.27–7.08%, within the phylum Acidobacteria) that have been found in coal bed methane production wells [84], methane hydrate-associated phylum Atribacteria (0.10 to 2.30%, formerly JS1/OP9) [85], seep-related sulfate-reducing bacteria *Desulfatiglans* [86], unknown phylogroups, like family Sva0485 (0.48 to 2.23%, within Deltaproteobacteria), the phylum Omnitrophicaeota (0.66 to 1.86%), etc. (Table S5).

### 3.7. Diverse Sulfate-Reducing Bacteria Were Found at the SYER Cold Seep

The known sulfate-reducing partners of AOM consortia with the ANME-1 clade have been identified as the HotSeep-1 cluster and SEEP-SRB2 cluster [8,9,10,87]. In the push core ORI-1163B-Dive91-Psc4, potential sulfate-reducing bacteria are abundant and diverse in three sampling depths, such as Desulfarculaceae (0.67–4.76%), Desulfobacteraceae (4.41–6.30%), Desulfobulbaceae (3.27–7.77%) and Desulfuromonadaceae (0.07–0.40%) (Table S5). Among them, the known SRB partners of ANMEs possess SEEP-SRB1 (0.86–2.58%), SEEP-SRB2 (0.03–5.70%), SEEP-SRB4 (0–0.20%), the genus *Desulfococcus* (0.00–0.17%), and *Desulfobulbus* (0.28–0.46%) (Table S5). Therefore, it was suggested that ANME-1b may perform the AOM in collaboration with ANME-2ab and Desulfarculaceae/Desulfobacteraceae/Desulfobulbaceae at the SYER cold seep.

### 3.8. The Prokaryotic Community of the SYER Cold Seep Is Distinct from Other SCS Cold Seeps

In previous microbial community studies at the SCS cold seeps, ANME-1b, the dominant archaeal phylotype in this study, also has high relative abundance at the cold seeps of Haima [29], Jiaolong [30] and Site GMGS2-08 [31], but it is not dominant at Haiyang [28]. ANME-2 groups, which exhibit minor relative abundance at the SYER cold seep, show less abundance than ANME-1 at Site GMGS2-08 and are not detected at the Haiyang cold seep but are predominant at the Haima and Jiaolong cold seeps. In addition, predominant SRB partners of AOM consortia are SEEP-SRB1 and SEEP-SRB2 at the cold seeps of SYER, Jiaolong and Site GMGS2-08. And some unique and dominant phylotypes, such as Halobacteriales at Haiyang and Hadesarchaea at Site GMGS2-08, were identified at different SCS cold seeps. Notably, high relative abundances of sulfur-oxidizing bacteria of *Sulfurimonas* and *Sulfurovum* were found at the SYER and Jiaolong cold seeps. Based on these comparisons, the microbial community of the SYER cold seep is distinct from other cold seeps studied at the SCS.

## 4. Conclusions

Here, we demonstrate the prokaryotic communities in and around the new species vestimentiferan tubeworm, *Paraescarpia formosa* ORI-1163B-Tw1, collected from the SYER cold seep offshore southwestern Taiwan at the northern SCS. The analysis of 16S rRNA gene clone library reveals that only one phylotype of sulfur-oxidizing endosymbionts which belonged to the family Sedimenticolaceae are found in the trunk of tubeworm *P. formosa* ORI-1163B-Tw1, which indicates that the endosymbionts in the trunk are monospecific sulfide-oxidizing bacteria. We also investigated archaeal and bacterial communities in the habitat of the tubeworm *P. formosa* ORI-1163B-Tw1 by using the method of the high-throughput amplicon sequencing of the (near) full-length 16S rRNA gene. The results showed that ANME-1b is the most dominant archaeal phylotype and ANME-2ab is minor in AOM consortia in the tubeworm’s habitat. The known sulfate-reducing partners of AOM consortia, such as SEEP-SRB1, SEEP-SRB2, and SEEP-SRB4 and genera *Desulfococcus* and *Desulfobulbus*, occur in small portions. It was suggested that ANME-1b may perform AOM in collaboration with ANME-2ab and Desulfarculaceae/Desulfobacteraceae/Desulfobulbaceae at the SYER cold seep. In addition, various potential methanogenic linages (Bathyarchaeota, Methanocellales, Methanomicrobiales, Methanosarcinales, Methanofastidiosales, and Methanomassiliicoccales) were detected at the microbial mat near the tubeworms’ habitat. Furthermore, although the temperature at the seabed of the SYER cold seep was not directly measured, it was estimated to be approximately 4 °C or lower. It is suggested that SYER cold seep communities, including methanogenic archaea, AOM consortia, tubeworms, and their symbionts, thrive in this cold environment. This study reveals the first investigation of microbiota in and around vestimentiferan tubeworms *P. formosa* discovered at the SYER cold seep offshore southwestern Taiwan at the northern SCS.

## Data Availability

The DDBJ/EMBL/GenBank accession numbers for the sequences obtained from clone libraries in this study are the tubeworm ORI-1163B-Tw1 COI gene (MH459063), 18S rRNA gene (MH464138), and 16S rRNA gene sequences of bacterial symbionts found in the axial rod (MH455345-MH455360), obturaculum (MH454643-MH454659) and trunk (MH454580-MH454596). The clean reads of 16S rRNA gene amplicon sequencing were submitted to the NCBI under BioProject PRJNA490800.

## Supplementary Materials

The following supporting information can be downloaded at: https://www.mdpi.com/article/10.3390/microorganisms12122452/s1, Figure S1: The map of spatial distribution offshore of southwestern Taiwan; Figure S2: Molecular phylogenetic analysis of the tubeworm ORI-1163B-Tw1 and related species based on 18S rRNA gene sequences; Figure S3: Phylogenetic analysis of SSU rRNA genes of bacterial symbionts from the trunk of tubeworm *P. formosa* ORI-1163B-Tw1 (labeled in red), other related sequences and the PacBio sequencing reads that belong to family Sedimenticolaceae in this study (labeled in brown); Figure S4: Vertical sulfate and sulfide concentration profiles in pore water in the sediments of the push core ORI-1163B-Dive91-Psc3; Figure S5: Phylogenetic tree of bathyarchaeotal 16S rRNA genes; Figure S6: Phylogenetic tree of 16S rRNA gene sequences of methanogens from this study and related strains or clones. Table S1: List of primers used in this study; Table S2: Statistics of PacBio 16S rRNA gene amplicon datasets analyzed by SILVAngs; Table S3: Statistics of PacBio 16S rRNA gene amplicon datasets analyzed by the EzBioCloud Apps of 16S-based MTP (Microbiome Taxonomic Profiling); Table S4: Detail archaeal distribution in push core ORI-1163B-Dive91-Psc4; Table S5: Detail bacterial distribution in push core ORI-1163B-Dive91-Psc4. References [37,65,88,89,90,91,92,93] are cited in the Supplementary Materials.
