# Supplementary material for: Microbial Communities in and Around the Siboglinid Tubeworms from the South Yungan East Ridge Cold Seep Offshore Southwestern Taiwan at the Northern South China Sea
Source: Microorganisms. 2024 Nov 28;12(12):2452. doi: 10.3390/microorganisms12122452 (PMC11676240; doi:10.3390/microorganisms12122452)
Supplement: Supplementary file 1 [file microorganisms-12-02452-s001.zip › microorganisms-3320175-supplementary.pdf]

Supplementary Materials

Microbial communities in and around the Siboglinid tubeworms from the South Yungan East Ridge cold seep offshore of southwestern Taiwan, at the northern South China Sea

Yin Li [1,2,3], Zhiwei Ye [1,2], Mei-Chin Lai [4,*], Char-Shine Liu [5], Charles K. Paull [6], Saulwood Lin [5], Shu-Jung Lai [4,7,8], Yi-Ting You [4], Sue-Yao Wu [4], Chuan-Chuan Hung [4], Jiun-Yan Ding [4], Chao-Jen Shih [9], Yen-Chi Wu [9], Jingjing Zhao [1,2,10], Wangchuan Xiao [1,2], Chih-Hung Wu [1,2,3], Guowen Dong [1,2,3], Hangying Zhang [1,2,11], Wanling Qiu [1,2,3], Song Wang [1,2,10] and Sheng-Chung Chen [1,2,3,4,10,*]

[1] School of Resources and Chemical Engineering, Sanming University, Sanming 365004, China; lijiang413508@126.com (Y.L.); 15294566095@163.com (Z.Y.); zhaojj191scus@163.com (J.Z.); xwc@fjsmu.edu.cn (W.X.); chihhung@yeah.net (C.-H.W.); gwdong2008@163.com (G.D.); 18005985018@163.com (H.Z.); wanning084020@163.com (W.Q.); 19154084327@163.com (S.W.)

[2] Fujian Provincial Key Laboratory of Resources and Environmental Monitoring and Sustainable
Management and Utilization, Sanming University, Sanming 365004, China

[3] College of Environment and Safety Engineering, Fuzhou University, Fuzhou 350108, China

[4] Department of Life Sciences, National Chung Hsing University, Taichung 402202, Taiwan; sjlai01@gmail.com (S.-J.L.); snoopy7117@gmail.com (Y.-T.Y.); sywu.kiki@gmail.com (S.-Y.W.); chuanchuanhung@gmail.com (C.-C.H.); jiunyanding@gmail.com (J.-Y.D.)

[5] Institute of Oceanography, National Taiwan University, Taipei 106319, Taiwan; csliu@ntu.edu.tw (C.-S.L.); swlin@ntu.edu.tw (S.L.)

[6] Monterey Bay Aquarium Research Institute, Moss Landing, CA 95039-9644, USA; paull@mbari.org

[7] Graduate Institute of Biomedical Sciences, China Medical University, Taichung 406040, Taiwan

[8] Research Center for Cancer Biology, China Medical University, Taichung 406040, Taiwan

[9] Bioresource Collection and Research Center, Food Industry Research and Development Institute, Hsinchu 300193, Taiwan; cjs23@firdi.org.tw (C.-J.S.); ycw@firdi.org.tw (Y.-C.W.)

[10] College of Chemistry and Materials Science, Fujian Normal University, Fuzhou 350117, China

[11] Medical Plant Exploitation and Utilization Engineering Research Center, Sanming University, Sanming 365004, China

* Correspondence: mclai01@gmail.com (M.-C.L.); benbear.xe@gmail.com (S.-C.C.)

5 supplementary Tables

6 supplementary Figures

Table S1. List of primers used in this study.

Target Gene	Primer name and sequence	Reference
For clone library		
18S rRNA gene	F-381: 5’-CCGGAGAGGGAGCCTGA-3’<br>R-1641: 5’-GGGCGGTGTGTACAAAGGG-3’	Katayama et al., 1996
COI gene	LCO-1490: 5’-GGTCAACAAATCATAAAGATATTGG-3’<br>HCO-2198: 5’-TAAACTTCAGGGTGACCAAAAAATCA-3’	Folmer et al., 1994
Bacterial 16S rRNA gene	8F: 5’-AGAGTTTGATCCTGGCTCAG-3’<br>1492RU: 5’-TTTTAATTAAGGTTACCTTGTTACGACTT-3’	Reysenbach et al., 1994<br>Gray and Herwig, 1996
For PacBio sequencing the near full-length 16S rRNA gene		
Archaeal 16S rRNA gene	21F(A):5’-TTCYGGTTGATCCYGCCGGA-3‘<br>1492R(16S_PacBio_V9R):5’-GGTTACCTTGTTACGACTT-3‘	Delong, 1992<br>Lane et al., 1991
Bacterial 16S rRNA gene	27F(B)(16S_Pacbio_V1F):5’-AGAGTTTGATCMTGGCTCAG-3‘<br>1492R(16S_PacBio_V9R):5’-GGTTACCTTGTTACGACTT-3‘	Lane et al., 1991

Table S2. Statistics of PacBio 16S rRNA gene amplicon datasets analyzed by SILVAngs[a].

Sample name[b]	Sequences	Avg. Length (bp)	Number of rejected sequences	Number of OTUs	Number of classified sequences	Number of "no relative"	Number of Archaea /Bacteria/Eukarya sequences
Dive91-Psc4_2-4cm_A	8,417	1,487	551 (6.55%)	2527	6918 (82.19%)	948 (11.26%)	5864/421/633
Dive91-Psc4_6-8cm_A	5,687	1,498	254 (4.47%)	2576	4707 (82.77%)	726 (12.77%)	4660/39/8
Dive91-Psc4_10-12cm_A	9,157	1,472	326 (3.56%)	2186	8405 (91.79%)	426 (4.65%)	8376/24/2
Dive91-Psc4_2-4cm_B	21,223	1,519	643 (3.03%)	10012	18854 (88.84%)	1726 (8.13%)	19/18828/7
Dive91-Psc4_6-8cm_B	3,469	1,543	167 (4.81%)	1909	2881 (83.05%)	421 (12.14%)	8/2872/1
Dive91-Psc4_10-12cm_B	8,922	1,535	317 (3.55%)	4095	7734 (86.68%)	871 (9.76%)	166/7568/0

[a]Software versions: SILVAngs 1.3; SINA v1.2.10 for ARB SVN (revision 21008); CD-Hit: 3.1.2; BLAST 2.2.30+.

[b]_A, using archaeal 16S rRNA primers; _B, using bacterial 16S rRNA primers.

Table S3. Statistics of PacBio 16S rRNA gene amplicon datasets analyzed by the EzBioCloud Apps of 16S-based MTP (Microbiome Taxonomic Profiling)[a].

Sample name[b]	Total reads after pre-filter	Avg. Length (bp)	Total valid reads	Number of reads identified at the species level	Number of species found
Dive91-Psc4_2-4cm_A	8,621	1,509.2	5,321 (61.7%)	3,658 (68.7%)	145
Dive91-Psc4_6-8cm_A	6,075	1,508.3	3,267 (53.8%)	2,896 (88.6%)	98
Dive91-Psc4_10-12cm_A	9,402	1,511.3	7,345 (78.1%)	7,019 (95.6%)	94
Dive91-Psc4_2-4cm_B	26,018	1,556.1	16,784 (64.5%)	13,753 (81.9%)	1,846
Dive91-Psc4_6-8cm_B	4,405	1,572.1	2,271 (51.6%)	2,050 (90.3%)	314
Dive91-Psc4_10-12cm_B	10,320	1571.7	6,263 (60.7%)	5,543 (88.5%)	498

[a]Database version, PKSSU4.0; region, V1V9. [b]_A, using archaeal 16S rRNA primers and target taxon is Archaea; _B, using bacterial 16S rRNA primers and target taxon is Bacteria.

Table S4. Detail archaeal distribution in push core ORI-1163B-Dive91-Psc4.

Taxonomy	2-4 cmbsf		6-8 cmbsf		10-12 cmbsf	
	Read#	Ratio (%)	Read#	Ratio (%)	Read#	Ratio (%)
Altiarchaeota;Altiarchaeia;	0	0.00	0	0.00	1	0.01
Asgardaeota;	1	0.02	0	0.00	0	0.00
Asgardaeota;Heimdallarchaeia;	14	0.24	2	0.04	0	0.00
Asgardaeota;Lokiarchaeia;	175	2.98	46	0.99	40	0.48
Asgardaeota;Odinarchaeia;	106	1.81	7	0.15	8	0.10
Crenarchaeota;Bathyarchaeia;	61	1.04	30	0.64	18	0.21
Diapherotrites;Iainarchaeia;	178	3.04	54	1.16	24	0.29
Diapherotrites;Micrarchaeia;	21	0.36	3	0.06	2	0.02
Euryarchaeota;Methanomicrobia;ANME-1;	0	0.00	0	0.00	1	0.01
Euryarchaeota;Methanomicrobia;ANME-1;ANME-1a;	34	0.58	27	0.58	21	0.25
Euryarchaeota;Methanomicrobia;ANME-1;ANME-1b;	461	7.86	2121	45.52	6488	77.46
Euryarchaeota;Methanomicrobia;Methanocellales;Methanocellaceae;Methanocella;	0	0.00	1	0.02	0	0.00
Euryarchaeota;Methanomicrobia;Methanocellales;Methanocellaceae;Rice Cluster I;	2	0.03	0	0.00	0	0.00
Euryarchaeota;Methanomicrobia;Methanocellales;uncultured;	26	0.44	16	0.34	7	0.08
Euryarchaeota;Methanomicrobia;Methanomicrobiales;Methanomicrobiaceae;Methanogenium;	1	0.02	0	0.00	0	0.00
Euryarchaeota;Methanomicrobia;Methanomicrobiales;Methanomicrobiaceae;uncultured;	0	0.00	0	0.00	1	0.01
Euryarchaeota;Methanomicrobia;Methanomicrobiales;Methanoregulaceae;Methanolinea;	1	0.02	0	0.00	0	0.00
Euryarchaeota;Methanomicrobia;Methanomicrobiales;Methanoregulaceae;Methanoregula;	3	0.05	2	0.04	1	0.01
Euryarchaeota;Methanomicrobia;Methanosarcinales;ANME-2a-2b;	105	1.79	155	3.33	180	2.15
Euryarchaeota;Methanomicrobia;Methanosarcinales;ANME-2a-2b;ANME-2b;	10	0.17	3	0.06	1	0.01
Euryarchaeota;Methanomicrobia;Methanosarcinales;ANME-2c;	23	0.39	44	0.94	34	0.41
Euryarchaeota;Methanomicrobia;Methanosarcinales;Methanoperedenaceae;	0	0.00	2	0.04	11	0.13
Euryarchaeota;Methanomicrobia;Methanosarcinales;Methanoperedenaceae;Candidatus Methanoperedens;	1	0.02	1	0.02	0	0.00
Euryarchaeota;Methanomicrobia;Methanosarcinales;Methanosaetaceae;Methanosaeta;	5	0.09	2	0.04	2	0.02
Euryarchaeota;Methanomicrobia;Methanosarcinales;Methanosarcinaceae;Methanococcoides;	72	1.23	299	6.42	164	1.96
Euryarchaeota;Methanomicrobia;Methanosarcinales;Methanosarcinaceae;Methanosarcina;	2	0.03	2	0.04	1	0.01
Euryarchaeota;Methanomicrobia;Methanosarcinales;Methermicoccaceae;	11	0.19	33	0.71	17	0.20
Euryarchaeota;Methanomicrobia;Methanosarcinales;Methermicoccaceae;Candidatus Syntrophoarchaeum;	1	0.02	0	0.00	1	0.01
Euryarchaeota;Thermococci;Methanofastidiosales;uncultured;	200	3.41	61	1.31	32	0.38
Euryarchaeota;Thermoplasmata;Marine Benthic Group D and DHVEG-1;	932	15.89	1034	22.19	772	9.22
Euryarchaeota;Thermoplasmata;Marine Group II;	3	0.05	1	0.02	1	0.01
Euryarchaeota;Thermoplasmata;Marine Group III;	10	0.17	0	0.00	0	0.00
Euryarchaeota;Thermoplasmata;Methanomassiliicoccales;Methanomethylophilaceae;uncultured;	0	0.00	0	0.00	1	0.01
Euryarchaeota;Thermoplasmata;Methanomassiliicoccales;uncultured;	278	4.74	66	1.42	14	0.17
Euryarchaeota;Thermoplasmata;SG8-5;	1379	23.52	211	4.53	164	1.96
Euryarchaeota;Thermoplasmata;uncultured;	1320	22.51	426	9.14	354	4.23
Hadesarchaeaeota;	5	0.09	3	0.06	1	0.01
Hydrothermarchaeota;	19	0.32	0	0.00	0	0.00
Nanoarchaeaeota;Nanohaloarchaeia;	0	0.00	1	0.02	0	0.00
Nanoarchaeaeota;Nanohaloarchaeia;Aenigmarchaeales;	2	0.03	1	0.02	2	0.02
Nanoarchaeaeota;Nanohaloarchaeia;Deep Sea Euryarchaeotic Group(DSEG);	23	0.39	2	0.04	8	0.10
Thaumarchaeota;Nitrososphaeria;Nitrosopumilales;Nitrosopumilaceae;	11	0.19	1	0.02	1	0.01
Thaumarchaeota;Nitrososphaeria;Nitrosopumilales;Nitrosopumilaceae;Candidatus Nitrosopumilus;	365	6.22	3	0.06	3	0.04
Thaumarchaeota;Nitrososphaeria;Nitrososphaerales;Nitrososphaeraceae;Candidatus Nitrososphaera;	3	0.05	0	0.00	0	0.00
Total	5864	100.00	4660	100.00	8376	100.00

Table S5. Detail bacterial distribution in push core ORI-1163B-Dive91-Psc4.

Taxonomy	2-4 cmbsf		6-8 cmbsf		10-12 cmbsf	
	Read#	Ratio (%)	Read#	Ratio (%)	Read#	Ratio (%)
Acidobacteria;Acidobacteriia;Acidobacteriales;Acidobacteriaceae (Subgroup 1);uncultured;	8	0.04	0	0.00	0	0.00
Acidobacteria;Acidobacteriia;Acidobacteriales;Koribacteraceae;Candidatus Koribacter;	24	0.13	0	0.00	2	0.03
Acidobacteria;Acidobacteriia;Acidobacteriales;uncultured;	88	0.47	1	0.03	5	0.07
Acidobacteria;Acidobacteriia;Solibacterales;Solibacteraceae (Subgroup 3);Bryobacter;	6	0.03	0	0.00	1	0.01
Acidobacteria;Acidobacteriia;Solibacterales;Solibacteraceae (Subgroup 3);Candidatus Solibacter;	4	0.02	0	0.00	1	0.01
Acidobacteria;Acidobacteriia;Solibacterales;Solibacteraceae (Subgroup 3);PAUC26f;	1	0.01	0	0.00	0	0.00
Acidobacteria;Acidobacteriia;Subgroup 13;	1	0.01	0	0.00	0	0.00
Acidobacteria;Acidobacteriia;Subgroup 2;	13	0.07	0	0.00	1	0.01
Acidobacteria;Aminicenantia;Aminicenantales;	50	0.27	71	2.47	329	4.35
Acidobacteria;AT-s3-28;	4	0.02	0	0.00	0	0.00
Acidobacteria;Blastocatellia (Subgroup 4);11-24;	5	0.03	0	0.00	1	0.01
Acidobacteria;Blastocatellia (Subgroup 4);Blastocatellales;Blastocatellaceae;Blastocatella;	2	0.01	0	0.00	0	0.00
Acidobacteria;Blastocatellia (Subgroup 4);Blastocatellales;Blastocatellaceae;Tellurimicrobium;	4	0.02	0	0.00	1	0.01
Acidobacteria;Blastocatellia (Subgroup 4);Blastocatellales;Blastocatellaceae;uncultured;	7	0.04	0	0.00	0	0.00
Acidobacteria;Blastocatellia (Subgroup 4);Pyrinomonadales;Pyrinomonadaceae;RB41;	7	0.04	0	0.00	2	0.03
Acidobacteria;c5LKS83;	0	0.00	0	0.00	4	0.05
Acidobacteria;d142;	1	0.01	0	0.00	0	0.00
Acidobacteria;Holophagae;Acanthopleuribacterales;Acanthopleuribacteraceae;Acanthopleuribacter;	6	0.03	1	0.03	1	0.01
Acidobacteria;Holophagae;Holophagales;Holophagaceae;Geothrix;	5	0.03	0	0.00	0	0.00
Acidobacteria;Holophagae;Subgroup 7;	44	0.23	0	0.00	0	0.00
Acidobacteria;Subgroup 11;	4	0.02	0	0.00	0	0.00
Acidobacteria;Subgroup 15;	1	0.01	0	0.00	0	0.00
Acidobacteria;Subgroup 17;	4	0.02	0	0.00	0	0.00
Acidobacteria;Subgroup 18;	21	0.11	0	0.00	0	0.00
Acidobacteria;Subgroup 21;	16	0.08	3	0.10	6	0.08
Acidobacteria;Subgroup 22;	99	0.53	0	0.00	5	0.07
Acidobacteria;Subgroup 25;	10	0.05	0	0.00	0	0.00
Acidobacteria;Subgroup 26;	2	0.01	0	0.00	1	0.01
Acidobacteria;Subgroup 5;	8	0.04	1	0.03	1	0.01
Acidobacteria;Subgroup 6;	46	0.24	1	0.03	4	0.05
Acidobacteria;Subgroup 6;Unknown Order;Unknown Family;Vicinamibacter;	0	0.00	0	0.00	1	0.01
Acidobacteria;Subgroup 9;	0	0.00	2	0.07	2	0.03
Acidobacteria;Thermoanaerobaculia;Thermoanaerobaculales;Thermoanaerobaculaceae;Subgroup 10;	16	0.08	0	0.00	2	0.03
Acidobacteria;Thermoanaerobaculia;Thermoanaerobaculales;Thermoanaerobaculaceae;Subgroup 23;	144	0.76	16	0.56	40	0.53
Acidobacteria;Thermoanaerobaculia;Thermoanaerobaculales;Thermoanaerobaculaceae;TPD-58;	7	0.04	0	0.00	3	0.04
Actinobacteria;Acidimicrobiia;Actinomarinales;Actinomarinaceae;Candidatus Actinomarina;	1	0.01	0	0.00	0	0.00
Actinobacteria;Acidimicrobiia;Actinomarinales;uncultured;	32	0.17	2	0.07	12	0.16
Actinobacteria;Acidimicrobiia;IMCC26256;	2	0.01	0	0.00	0	0.00
Actinobacteria;Acidimicrobiia;Microtrichales;Iamiaceae;Iamia;	1	0.01	0	0.00	0	0.00
Actinobacteria;Acidimicrobiia;Microtrichales;Ilumatobacteraceae;Ilumatobacter;	3	0.02	0	0.00	0	0.00
Actinobacteria;Acidimicrobiia;Microtrichales;Ilumatobacteraceae;uncultured;	3	0.02	1	0.03	0	0.00
Actinobacteria;Acidimicrobiia;Microtrichales;Microtrichaceae;Sva0996 marine group;	4	0.02	0	0.00	0	0.00
Actinobacteria;Acidimicrobiia;Microtrichales;uncultured;	5	0.03	0	0.00	0	0.00
Actinobacteria;Acidimicrobiia;uncultured;	1	0.01	0	0.00	0	0.00
Actinobacteria;Actinobacteria;Catenulisporales;Catenulisporaceae;Catenulispora;	1	0.01	0	0.00	0	0.00
Actinobacteria;Actinobacteria;Corynebacteriales;Mycobacteriaceae;Mycobacterium;	3	0.02	0	0.00	0	0.00
Actinobacteria;Actinobacteria;Corynebacteriales;Nocardiaceae;Nocardia;	1	0.01	0	0.00	0	0.00
Actinobacteria;Actinobacteria;Frankiales;Acidothermaceae;Acidothermus;	2	0.01	0	0.00	0	0.00
Actinobacteria;Actinobacteria;Frankiales;Frankiaceae;Jatrophihabitans;	2	0.01	0	0.00	0	0.00
Actinobacteria;Actinobacteria;Micrococcales;Intrasporangiaceae;Intrasporangium;	1	0.01	0	0.00	0	0.00
Actinobacteria;Actinobacteria;Micrococcales;Intrasporangiaceae;Oryzihumus;	1	0.01	0	0.00	0	0.00
Actinobacteria;Actinobacteria;Micrococcales;Intrasporangiaceae;Phycicoccus;	2	0.01	0	0.00	0	0.00
Actinobacteria;Actinobacteria;Micrococcales;Intrasporangiaceae;Tetrasphaera;	1	0.01	0	0.00	0	0.00
Actinobacteria;Actinobacteria;Micrococcales;Microbacteriaceae;uncultured;	1	0.01	0	0.00	0	0.00
Actinobacteria;Actinobacteria;Micromonosporales;Micromonosporaceae;Hamadaea;	0	0.00	0	0.00	2	0.03
Actinobacteria;Actinobacteria;Micromonosporales;Micromonosporaceae;Micromonospora;	2	0.01	0	0.00	0	0.00
Actinobacteria;Actinobacteria;Propionibacteriales;Nocardioidaceae;Nocardioides;	1	0.01	1	0.03	0	0.00
Actinobacteria;Actinobacteria;Pseudonocardiales;Pseudonocardiaceae;Pseudonocardia;	1	0.01	0	0.00	0	0.00
Actinobacteria;Actinobacteria;Streptomycetales;Streptomycetaceae;Streptomyces;	4	0.02	0	0.00	0	0.00
Actinobacteria;Actinobacteria;Streptosporangiales;Streptosporangiaceae;Microbispora;	1	0.01	0	0.00	0	0.00
Actinobacteria;Actinobacteria;Streptosporangiales;Streptosporangiaceae;Nonomuraea;	1	0.01	0	0.00	0	0.00
Actinobacteria;Coriobacteriia;OPB41;	2	0.01	0	0.00	0	0.00
Actinobacteria;MB-A2-108;	3	0.02	0	0.00	0	0.00
Actinobacteria;RBG-16-55-12;	0	0.00	0	0.00	1	0.01
Actinobacteria;Thermoleophilia;Gaiellales;Gaiellaceae;Gaiella;	15	0.08	0	0.00	0	0.00
Actinobacteria;Thermoleophilia;Gaiellales;uncultured;	17	0.09	0	0.00	0	0.00
Actinobacteria;Thermoleophilia;Solirubrobacterales;67-14;	3	0.02	0	0.00	0	0.00
Actinobacteria;Thermoleophilia;Solirubrobacterales;Solirubrobacteraceae;Conexibacter;	2	0.01	0	0.00	0	0.00
Actinobacteria;WCHB1-81;	4	0.02	3	0.10	8	0.11
Aegiribacteria;	33	0.18	6	0.21	16	0.21
AncK6;	3	0.02	4	0.14	4	0.05
Armatimonadetes;Armatimonadia;Armatimonadales;	1	0.01	0	0.00	0	0.00
Armatimonadetes;Chthonomonadetes;Chthonomonadales;	7	0.04	1	0.03	0	0.00
Armatimonadetes;Chthonomonadetes;Chthonomonadales;Chthonomonadaceae;Chthonomonas;	1	0.01	0	0.00	0	0.00
Armatimonadetes;DG-56;	1	0.01	3	0.10	2	0.03
Armatimonadetes;Fimbriimonadia;Fimbriimonadales;Fimbriimonadaceae;	10	0.05	0	0.00	1	0.01
Armatimonadetes;uncultured;	4	0.02	1	0.03	3	0.04

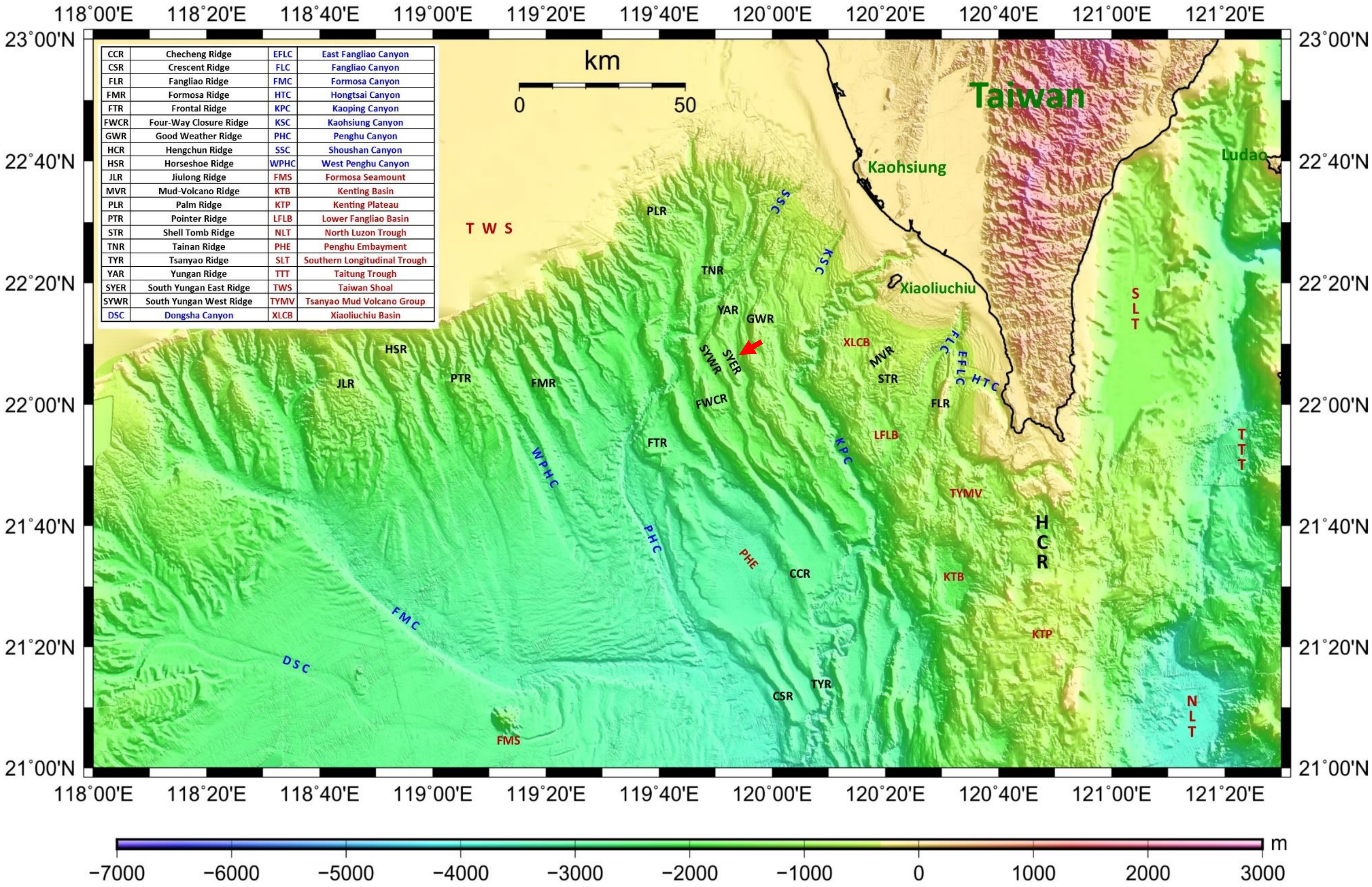

Figure S1. The map of spatial distribution offshore of southwestern Taiwan. The red arrow indicates the region of the South Yungan East Ridge (SYER) in this study. This figure was modified from Chung et al. (2016).

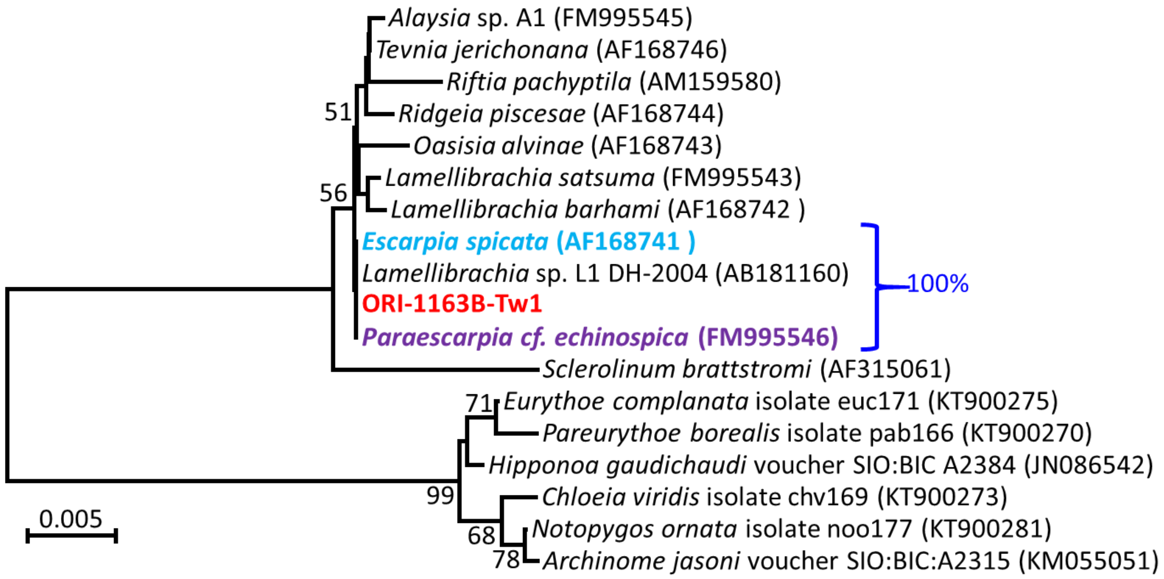

Figure S2. Molecular phylogenetic analysis of the tubeworm ORI-1163B-Tw1 and related species based on 18S rRNA gene sequences. The evolutionary history was inferred by using the Neighbor Joining method and evolutionary analyses were conducted in MEGA7 (Kumar et al., 2016). Numbers at the nodes indicate the proportion of occurrences in 1000 bootstrap replicates. The scale represents 0.005 substitutions per nucleotide site.

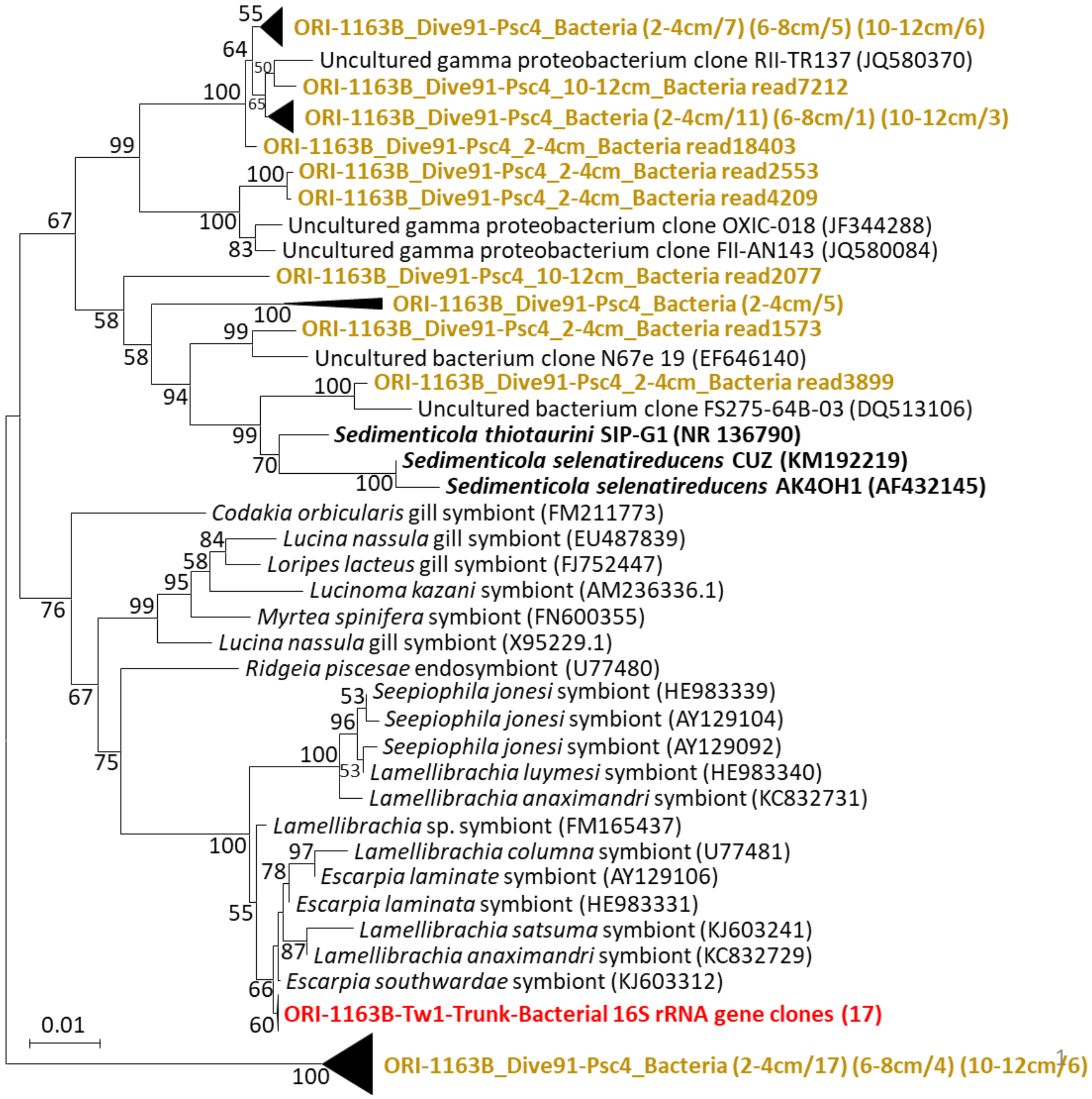

Figure S3. Phylogenetic analysis of SSU rRNA genes of bacterial symbionts from the trunk of tubeworm *P. formosa* ORI-1163B-Tw1 (labeled in red), other related sequences and the PacBio sequencing reads that belong to family Sedimenticolaceae in this study (labeled in brown). GenBank accession numbers or sampling depth/sequence read numbers are shown in parentheses. The cultivated representatives of genus *Sedimenticola* were labeled in bold. The evolutionary history was inferred by using the Neighbor-Joining method and evolutionary analyses were conducted in MEGA7 (Kumar et al., 2016). Numbers at the nodes indicate the proportion of occurrences in 1000 bootstrap replicates. The scale represents 0.01 substitutions per nucleotide site.

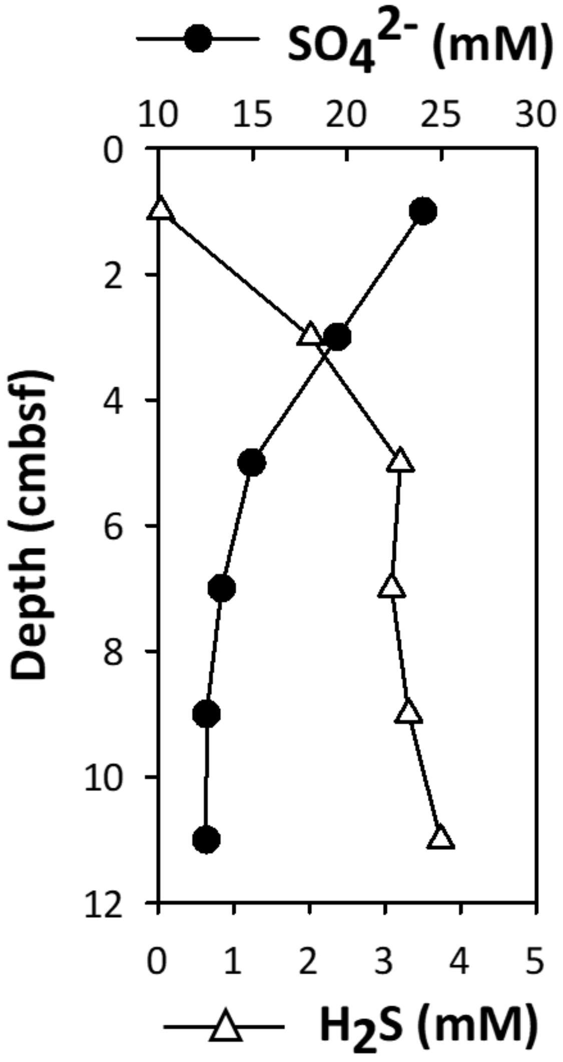

Figure S4. Vertical sulfate and sulfide concentration profiles in pore water in the sediments of the push core ORI-1163B-Dive91-Psc3.

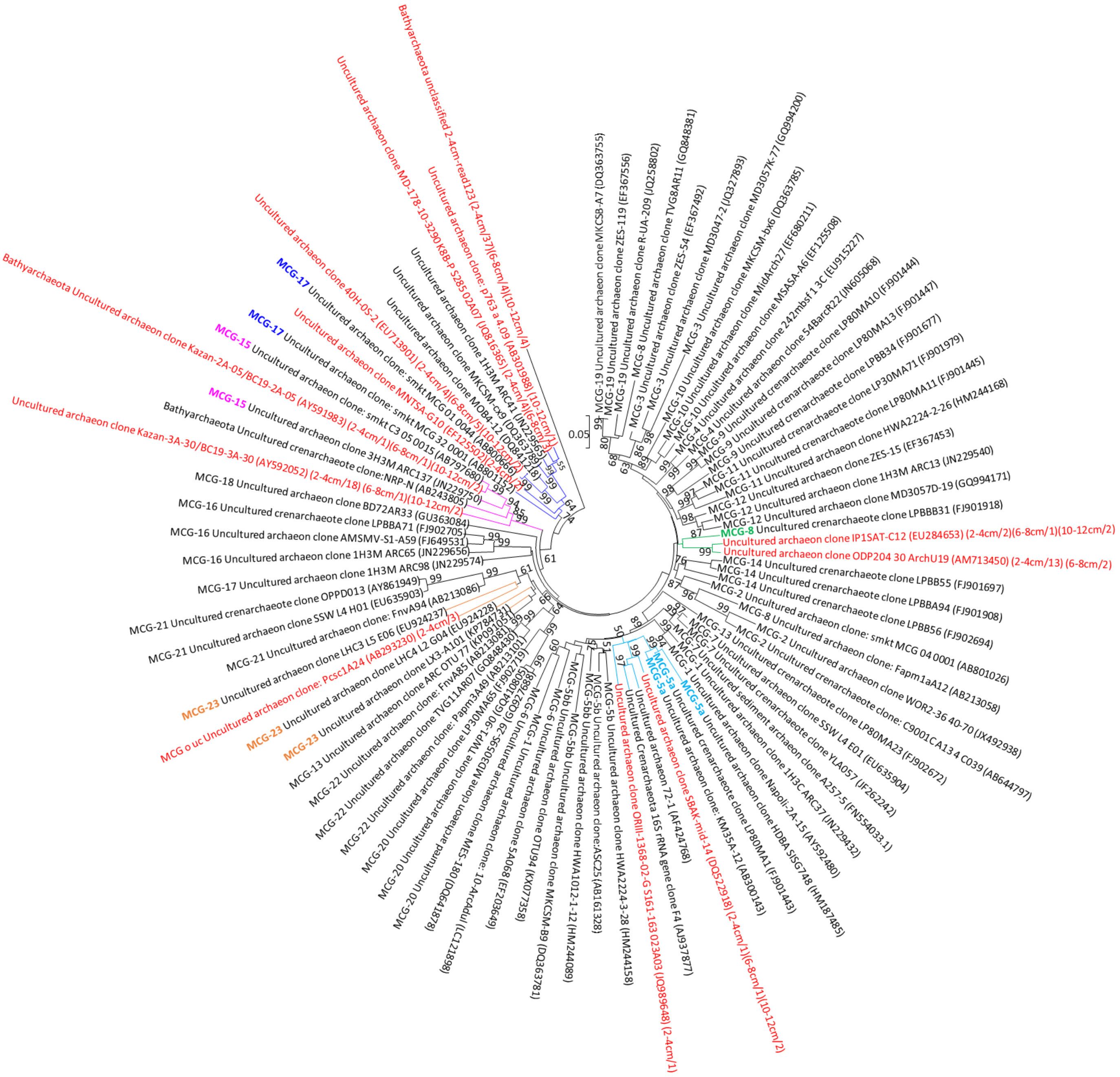

Figure S5. Phylogenetic tree of bathyarchaeotal 16S rRNA genes. The Miscellaneous Crenarchaeota group (MCG) subgroups assigned sequences were obtained from a previous review article (Zhou et al., 2018). GenBank accession numbers or sampling depth/sequence read numbers are shown in parentheses. The single sequence labeled in red is selected as a representative by clustering read sequences within 97% sequence similarity threshold. The evolutionary history was inferred by using the Neighbor-Joining method and evolutionary analyses were conducted in MEGA7 (Kumar et al., 2016). Bootstrap values at the nodes are percentages of 1000 replicates. Bar, 0.05 evolutionary distances.

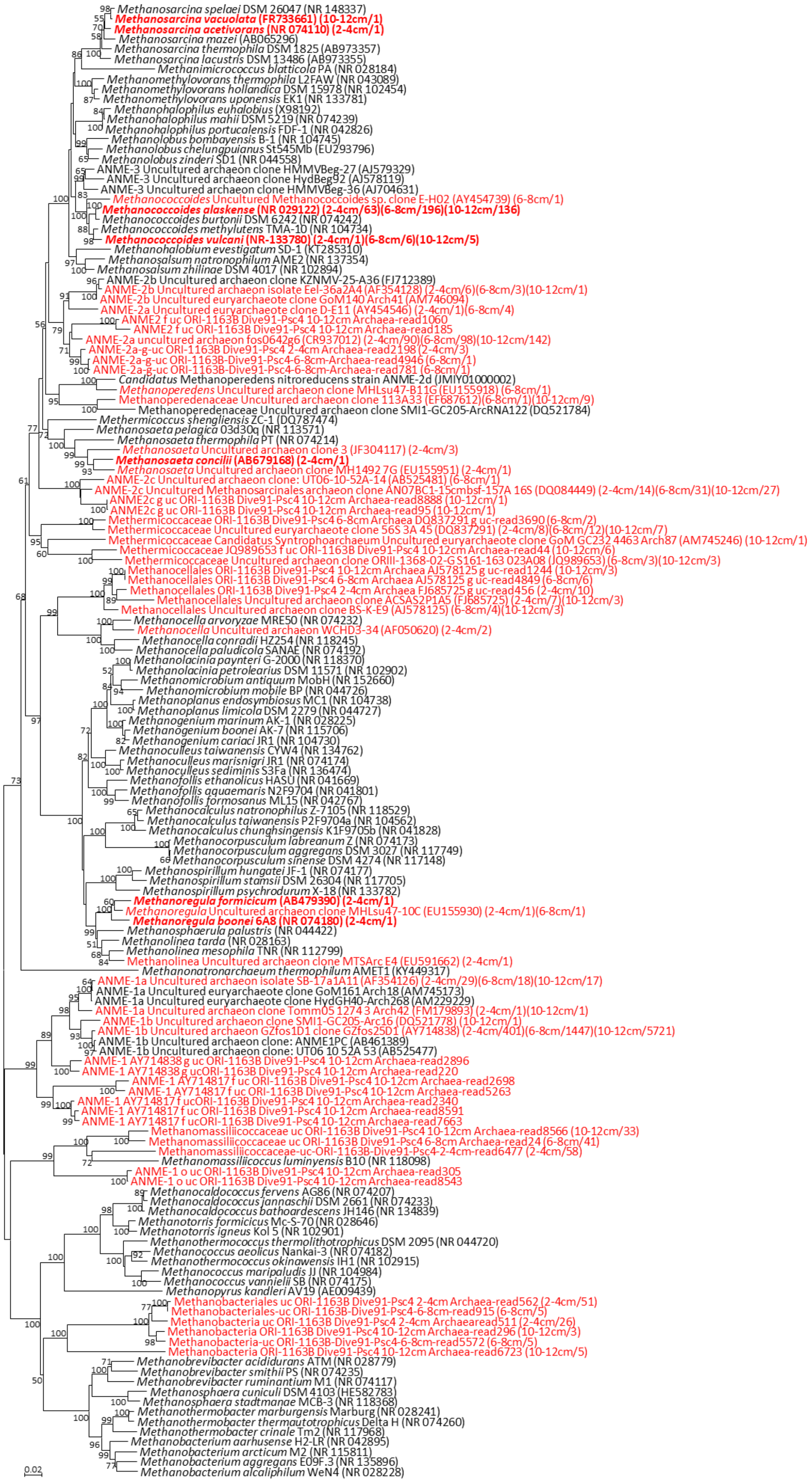
Methanosarcina spelaei DSM 26047 (NR 148337)
Methanosarcina vacuolata (FR733661) (10-12cm/1)
Methanosarcina acetivorans (NR 074110) (2-4cm/1)
Methanosarcina mazei (AB065296)
Methanosarcina thermophila DSM 1825 (AB973357)
Methanosarcina lacustris DSM 13486 (AB973355)
Methanimicrococcus blatticola PA (NR 028184)
Methanomethylovorans thermophila L2FAW (NR 043089)
Methanomethylovorans hollandica DSM 15978 (NR 102454)
Methanomethylovorans uponensis EK1 (NR 133781)
Methanohalophilus euhalobius (X98192)
Methanohalophilus mahii DSM 5219 (NR 074239)
Methanohalophilus portucalensis FDF-1 (NR 042826)
Methanolobus bombayensis B-1 (NR 104745)
Methanolobus chelungpuianus St545Mb (EU293796)
Methanolobus zinderi SD1 (NR 044558)
ANME-3 Uncultured archaeon clone HMMVBeg-27 (AJ579329)
ANME-3 Uncultured archaeon clone HydBeg92 (AJ578119)
ANME-3 Uncultured archaeon clone HMMVBeg-36 (AJ704631)
Methanococcoides Uncultured Methanococcoides sp. clone E-H02 (AY454739) (6-8cm/1)
Methanococcoides alaskense (NR 029122) (2-4cm/63)(6-8cm/196)(10-12cm/136)
Methanococcoides burtonii DSM 6242 (NR 074242)
Methanococcoides methylutens TMA-10 (NR 104734)
Methanococcoides vulcani (NR-133780) (2-4cm/1)(6-8cm/6)(10-12cm/5)
Methanohalobium evestigatum SD-1 (KT285310)
Methanosalsum natronophilum AME2 (NR 137354)
Methanosalsum zhilinae DSM 4017 (NR 102894)
ANME-2b Uncultured archaeon clone KZNMV-25-A36 (FJ712389)
ANME-2b Uncultured archaeon isolate Eel-36a2A4 (AF354128) (2-4cm/6)(6-8cm/3)(10-12cm/1)
ANME-2b Uncultured euryarchaeote clone GoM140 Arch41 (AM746094)
ANME-2a Uncultured euryarchaeote clone D-E11 (AY454546) (2-4cm/1)(6-8cm/4)
ANME2 f uc ORI-1163B Dive91-Psc4 10-12cm Archaea-read1060
ANME2 f uc ORI-1163B Dive91-Psc4 10-12cm Archaea-read185
ANME-2a uncultured archaeon fos0642g6 (CR937012) (2-4cm/90)(6-8cm/98)(10-12cm/142)
ANME-2a-g-uc ORI-1163B Dive91-Psc4 2-4cm Archaea-read2198 (2-4cm/3)
ANME-2a-g-uc ORI-1163B-Dive91-Psc4-6-8cm-Archaea-read4946 (6-8cm/1)
ANME-2a-g-uc ORI-1163B-Dive91-Psc4-6-8cm-Archaea-read781 (6-8cm/1)
Candidatus Methanoperedens nitroreducens strain ANME-2d (JMIY01000002)
Methanoperedens Uncultured archaeon clone MHLsu47-B11G (EU155918) (6-8cm/1)
Methanoperedenaceae Uncultured archaeon clone 113A33 (EF687612)(6-8cm/1)(10-12cm/9)
Methanoperedenaceae Uncultured archaeon clone SMI1-GC205-ArcRNA122 (DQ521784)
Methermicoccus shengliensis ZC-1 (DQ787474)
Methanosaeta pelagica 03d30q (NR 113571)
Methanosaeta thermophila PT (NR 074214)
Methanosaeta Uncultured archaeon clone 3 (JF304117) (2-4cm/3)
Methanosaeta concilii (AB679168) (2-4cm/1)
Methanosaeta Uncultured archaeon clone MH1492 7G (EU155951) (2-4cm/1)
ANME-2c Uncultured archaeon clone: UT06-10-52A-14 (AB525481) (6-8cm/1)
ANME-2c Uncultured Methanosarcinales archaeon clone AN07BC1-15cmbsf-157A 16S (DQ084449) (2-4cm/14)(6-8cm/31)(10-12cm/27)
ANME2c g uc ORI-1163B Dive91-Psc4 10-12cm Archaea-read8888 (10-12cm/1)
ANME2c g uc ORI-1163B Dive91-Psc4 10-12cm Archaea-read95 (10-12cm/1)
Methermicoccaceae ORI-1163B Dive91-Psc4 6-8cm Archaea DQ837291 g uc-read3690 (6-8cm/2)
Methermicoccaceae Uncultured euryarchaeote clone 56S 3A 45 (DQ837291) (2-4cm/8)(6-8cm/12)(10-12cm/7)
Methermicoccaceae Candidatus Syntrophoarchaeum Uncultured euryarchaeote clone GoM GC232 4463 Arch87 (AM745246) (10-12cm/1)
Methermicoccaceae JQ989653 f uc ORI-1163B Dive91-Psc4 10-12cm Archaea-read44 (10-12cm/6)
Methermicoccaceae Uncultured archaeon clone ORIII-1368-02-G S161-163 023A08 (JQ989653) (6-8cm/3)(10-12cm/3)
Methanocellales ORI-1163B Dive91-Psc4 10-12cm Archaea AJ578125 g uc-read1244 (10-12cm/3)
Methanocellales ORI-1163B Dive91-Psc4 6-8cm Archaea AJ578125 g uc-read4849 (6-8cm/6)
Methanocellales ORI-1163B Dive91-Psc4 2-4cm Archaea FJ685725 g uc-read456 (2-4cm/10)
Methanocellales Uncultured archaeon clone ACSAS2P1A5 (FJ685725) (2-4cm/7)(10-12cm/3)
Methanocellales Uncultured archaeon clone BS-K-E9 (AJ578125) (6-8cm/4)(10-12cm/3)
Methanocella arvoryzae MRE50 (NR 074232)
Methanocella Uncultured archaeon WCHD3-34 (AF050620) (2-4cm/2)
Methanocella conradii HZ254 (NR 118245)
Methanocella paludicola SANAE (NR 074192)
Methanolacinia paynteri G-2000 (NR 118370)
Methanolacinia petrolearius DSM 11571 (NR 102902)
Methanomicrobium antiquum MobH (NR 152660)
Methanomicrobium mobile BP (NR 044726)
Methanoplanus endosymbiosus MC1 (NR 104738)
Methanoplanus limicola DSM 2279 (NR 044727)
Methanogenium marinum AK-1 (NR 028225)
Methanogenium boonei AK-7 (NR 115706)
Methanogenium cariaci JR1 (NR 104730)
Methanoculleus taiwanensis CYW4 (NR 134762)
Methanoculleus marisnigri JR1 (NR 074174)
Methanoculleus sediminis S3Fa (NR 136474)
Methanofollis ethanolicus HASU (NR 041669)
Methanofollis aquaemaris N2F9704 (NR 041801)
Methanofollis formosanus ML15 (NR 042767)
Methanocalculus natronophilus Z-7105 (NR 118529)
Methanocalculus taiwanensis P2F9704a (NR 104562)
Methanocalculus chunghsingensis K1F9705b (NR 041828)
Methanocorpusculum labreanum Z (NR 074173)
Methanocorpusculum aggregans DSM 3027 (NR 117749)
Methanocorpusculum sinense DSM 4274 (NR 117148)
Methanospirillum hungatei JF-1 (NR 074177)
Methanospirillum stamsii DSM 26304 (NR 117705)
Methanospirillum psychrodurum X-18 (NR 133782)
Methanoregula formicicum (AB479390) (2-4cm/1)
Methanoregula Uncultured archaeon clone MHLsu47-10C (EU155930) (2-4cm/1)(6-8cm/1)
Methanoregula boonei 6A8 (NR 074180) (2-4cm/1)
Methanosphaerula palustris (NR 044422)
Methanolinea tarda (NR 028163)
Methanolinea mesophila TNR (NR 112799)
Methanolinea Uncultured archaeon clone MTSArc E4 (EU591662) (2-4cm/1)
Methanonatronarchaeum thermophilum AMET1 (KY449317)
ANME-1a Uncultured archaeon isolate SB-17a1A11 (AF354126) (2-4cm/29)(6-8cm/18)(10-12cm/17)
ANME-1a Uncultured euryarchaeote clone GoM161 Arch18 (AM745173)
ANME-1a Uncultured euryarchaeote clone HydGH40-Arch268 (AM229229)
ANME-1a Uncultured archaeon clone Tomm05 1274 3 Arch42 (FM179893) (2-4cm/1)(10-12cm/1)
ANME-1b Uncultured archaeon clone SMI1-GC205-Arc16 (DQ521778) (10-12cm/1)
ANME-1b Uncultured archaeon GZfos1D1 clone GZfos25D1 (AY714838) (2-4cm/401)(6-8cm/1447)(10-12cm/5721)
ANME-1b Uncultured archaeon clone: ANME1PC (AB461389)
ANME-1b Uncultured archaeon clone: UT06 10 52A 53 (AB525477)
ANME-1 AY714838 g uc ORI-1163B Dive91-Psc4 10-12cm Archaea-read2896
ANME-1 AY714838 g ucORI-1163B Dive91-Psc4 10-12cm Archaea-read220
ANME-1 AY714817 f uc ORI-1163B Dive91-Psc4 10-12cm Archaea-read2698
ANME-1 AY714817 f uc ORI-1163B Dive91-Psc4 10-12cm Archaea-read5263
ANME-1 AY714817 f ucORI-1163B Dive91-Psc4 10-12cm Archaea-read2340
ANME-1 AY714817 f uc ORI-1163B Dive91-Psc4 10-12cm Archaea-read8591
ANME-1 AY714817 f ucORI-1163B Dive91-Psc4 10-12cm Archaea-read7663
Methanomassiliicoccaceae uc ORI-1163B Dive91-Psc4 10-12cm Archaea-read8566 (10-12cm/33)
Methanomassiliicoccaceae uc ORI-1163B Dive91-Psc4 6-8cm Archaea-read24 (6-8cm/41)
Methanomassiliicoccaceae-uc-ORI-1163B-Dive91-Psc4-2-4cm-read6477 (2-4cm/58)
Methanomassiliicoccus luminyensis B10 (NR 118098)
ANME-1 o uc ORI-1163B Dive91-Psc4 10-12cm Archaea-read305
ANME-1 o uc ORI-1163B Dive91-Psc4 10-12cm Archaea-read8543
Methanocaldococcus fervens AG86 (NR 074207)
Methanocaldococcus jannaschii DSM 2661 (NR 074233)
Methanocaldococcus bathoardescens JH146 (NR 134839)
Methanotorris formicicus Mc-S-70 (NR 028646)
Methanotorris igneus Kol 5 (NR 102901)
Methanothermococcus thermolithotrophicus DSM 2095 (NR 044720)
Methanococcus aeolicus Nankai-3 (NR 074182)
Methanothermococcus okinawensis IH1 (NR 102915)
Methanococcus maripaludis JJ (NR 104984)
Methanococcus vannielii SB (NR 074175)
Methanopyrus kandleri AV19 (AE009439)
Methanobacteriales uc ORI-1163B Dive91-Psc4 2-4cm Archaea-read562 (2-4cm/51)
Methanobacteriales-uc ORI-1163B-Dive91-Psc4-6-8cm-read915 (6-8cm/5)
Methanobacteria uc ORI-1163B Dive91-Psc4 2-4cm Archaearead511 (2-4cm/26)
Methanobacteria ORI-1163B Dive91-Psc4 10-12cm Archaea-read296 (10-12cm/3)
Methanobacteria-uc ORI-1163B-Dive91-Psc4-6-8cm-read5572 (6-8cm/5)
Methanobacteria ORI-1163B Dive91-Psc4 10-12cm Archaea-read6723 (10-12cm/5)
Methanobrevibacter acididurans ATM (NR 028779)
Methanobrevibacter smithii PS (NR 074235)
Methanobrevibacter ruminantium M1 (NR 074117)
Methanosphaera cuniculi DSM 4103 (HE582783)
Methanosphaera stadtmanae MCB-3 (NR 118368)
Methanothermobacter marburgensis Marburg (NR 028241)
Methanothermobacter thermautotrophicus Delta H (NR 074260)
Methanothermobacter crinale Tm2 (NR 117968)
Methanobacterium aarhusense H2-LR (NR 042895)
Methanobacterium arcticum M2 (NR 115811)
Methanobacterium aggregans E09F.3 (NR 135896)
Methanobacterium alcaliphilum WeN4 (NR 028228)
0.02

Figure S6. Phylogenetic tree of 16S rRNA gene sequences of methanogens from this study and related strains or clones. GenBank accession numbers or sampling depth range/sequence read numbers are shown in parentheses. The single sequence labeled in red is selected as a representative by clustering read sequences within 97% sequence similarity threshold. The read sequences matched (>97% similarity) to known and cultured strains are labeled in bold. The evolutionary history was inferred by using the Neighbor-Joining method and evolutionary analyses were conducted in MEGA7 (Kumar et al., 2016). Bootstrap values at the nodes are percentages of 1000 replicates. Bar, 0.02 evolutionary distances.

Supplementary references

1. Chung, S.H., T.S. Lin, C.C. Lin, C.S. Liu, S.C. Chen, Y.S. Wang, C.Y. Wei, and P.C. Chen. 2016. Geological investigation of gas hydrate resource potential in the offshore areas of south-western Taiwan. Special Publication of the Central Geological Survey. 30:1-42.
2. Folmer, O., M. Black, W. Hoeh, R. Lutz, and R. Vrijenhoek. 1994. DNA primers for amplification of mitochondrial cytochrome c oxidase subunit I from diverse metazoan invertebrates. Mol. Mar. Biol. Biotechnol. 3:294-299.
3. Gray, J.P., and R.P. Herwig. 1996. Phylogenetic analysis of the bacterial communities in marine sediments. Appl. Environ. Microbiol. 62:4049-4059.
4. Katayama, T., M. Nishioka, and M. Yamamoto. 1996. Phylogenetic relationships among turbellarian orders inferred from 18S rDNA sequences. Zoolog Sci. 13:747-756.
5. Kumar, S., G. Stecher, and K. Tamura. 2016. MEGA7: Molecular Evolutionary Genetics Analysis Version 7.0 for Bigger Datasets. Mol. Biol. Evol. 33:1870-1874.
6. Lane, D.J. 1991. 16S/23S rRNA sequencing. In: Nucleic acid techniques in bacterial systematics. Stackebrandt, E., and Goodfellow, M., eds., John Wiley and Sons, New York, NY, pp. 115-175.
7. Reysenbach, A.L., G.S. Wickham, and N.R. Pace. 1994. Phylogenetic analysis of the hyperthermophilic pink filament community in Octopus Spring, Yellowstone National Park. Appl. Environ. Microbiol. 60:2113-2119.
8. Zhou, Z.C., J. Pan, F.P. Wang, J.D. Gu, and M. Li. 2018. Bathyarchaeota: globally distributed metabolic generalists in anoxic environments. FEMS Microbiol. Rev. 42:639-655.
